# Risk Factors for Recurrent Arterial Ischemic Stroke in Children and Young Adults

**DOI:** 10.3390/brainsci10010024

**Published:** 2020-01-02

**Authors:** Beata Sarecka-Hujar, Ilona Kopyta

**Affiliations:** 1 Department of Pharmaceutical Technology, School of Pharmacy with the Division of Laboratory Medicine in Sosnowiec, Medical University of Silesia in Katowice, 41-200 Sosnowiec, Poland; 2 Department of Paediatric Neurology, School of Medicine in Katowice, Medical University of Silesia in Katowice, 40-752 Katowice, Poland; ilonakopyta@autograf.pl

**Keywords:** arterial ischemic stroke (AIS), recurrent stroke, recurrence, children, young adults, risk factors

## Abstract

Arterial ischemic stroke (AIS) experienced at a young age is undoubtedly a serious medical problem. AIS very rarely occurs at a developmental age, whereas in young adults, it occurs with a higher frequency. The etiologic mechanisms of AIS occurring in childhood and adulthood differ. However, for both age populations, neurological consequences of AIS, including post-stroke seizures, motor disability, and recurrence of the disease, are connected to many years of care, rehabilitation, and treatment. Recurrent stroke was observed to increase the risk of patients’ mortality. One of the confirmed risk factors for recurrent stroke in children is the presence of vasculopathies, especially Moyamoya disease and syndrome, and focal cerebral arteriopathy of childhood (FCA). FCA causes a 5-fold increase in the risk of recurrent stroke in comparison with idiopathic AIS. In turn, young adults with recurrent stroke were found to more often suffer from hypertension, diabetes mellitus, or peripheral artery disease than young patients with first-ever stroke. Some reports also indicate relationships between specific genetic polymorphisms and AIS recurrence in both age groups. The aim of the present literature review was to discuss available data regarding the risk factors for recurrent AIS in children and young adults.

## 1. Introduction

Arterial ischemic stroke occurring in children and young adults is a serious medical problem. Taking into account the fact that children and young adults who survive AIS will live longer than older stroke patients, it is important to optimize the medical management for these patients and properly recognize impairments in cognition and mood, because they can be significant barriers in patients’ independent life. The incidence rate for pediatric AIS is about 1.2 to 7.9/100,000 children per year [1,2]. The differences result from ethnic and genetic variability, as well as the age range of the populations examined.

Most researchers analysing pediatric AIS exclude newborns. Therefore, the age of pediatric patients with AIS ranges from 29 days of life to 18 years [3,4,5,6]. In a group of young adults, the prevalence of AIS varies from 3.4 to 11.3/100,000 persons per year [7,8]. On the other hand, Groppo et al. [9] estimated the mean annual incidence rate of first-ever stroke in young adults to be 12.1 cases per 100,000 person-years in an Italian population from Ferrara. It was demonstrated that in a group of adult patients with AIS, 10% to 15% of all strokes were experienced by young adults [10,11]. The data also indicate different percentages of stroke frequency in young adults and adolescents compared to all strokes; a study performed in the USA showed that 5% of all strokes occurred in a young adult population aged between 18 and 44 years [12]. A particularly high prevalence of AIS in young adults was previously observed in developing countries. However, the results were based on a small number of young patients with AIS [13].

The wide range of stroke incidence in young adults may result from the heterogeneity of individual patient populations, as well as contradictions in defining a person as a “young adult”. The available data may be confusing in terms of this matter. In the literature, a person between the age of 18 and 45 is considered to be a young adult. The upper age limit has been extended to 50 years by some authors [14,15]. In turn, Kissela et al. [16] considered people under the age of 54 as younger adults. A similar upper age limit of 55 years was adopted by Ferro et al. [17]. Surprisingly, all stroke patients aged 15 to 60 with radiologically confirmed AIS were recruited as part of The Norwegian Stroke in the Young Study [18]. In turn, according to Prasal and Singhal, studies on stroke in young people should consider cases for those aged 15–49 years (<50 years) [19] since AIS risk factors for people older than 50 years are usually similar to those present in the elderly. As for the lower age limit, 15-year-old patients are often analyzed together with pediatric patients.

The etiologic mechanisms of AIS occurring in adulthood and at a developmental age differ. Similarity may be found in the case of a significant predominance of the male sex, both in pediatric patients with AIS and in young adults with stroke [20,21,22]. However, some discrepancies have also been noted in this respect as Rasura et al. [23] observed higher incidence rates for stroke in young females than in young males (8.4 vs. 7.6) among stroke patients from Rome. On the other hand, a reliable age-related analysis of 25 studies concerning the incidence of AIS in the young proved the predominance of the male sex in the presence of stroke [24]. The authors analyzed the following age groups: 0–14 years, 15–24 years, 25–34 years, and 35–44  years, obtaining the incidence rates of AIS ranging from 0.99 to 30.66 for men and from 0.73 to 23.99 for women, depending on age. The most important risk factors for AIS identified in children are arteriopathies, and congenital and acquired cardiac defects leading to cardioembolic stroke, as well as thrombophilia and prothrombotic states [25,26,27]. In turn, the etiologic factors for AIS in young adults include, among others, cardiac embolism, cervical artery dissection, atherothrombosis, and small vessel disease, whereas in almost 30% of cases, the cause of stroke is undetermined [28].

Despite this, the neurological consequences of AIS, including post-stroke seizures, motor disability, and recurrence of the disease, are detrimental for both age populations. Additionally, they involve huge costs related to many years of care, rehabilitation, and treatment.

According to the latest definition provided by deVeber et al. [29], recurrent stroke is defined as clinically symptomatic AIS events manifested as acute focal neurological deficits with infarction in a vascular distribution in neuroimaging, which began more than 24 h following the onset of the first stroke. The recurrence of stroke was demonstrated as a factor increasing the risk of mortality in patients. Aarnio et al. [30] observed particularly high mortality among stroke patients who experienced a recurrence of the disease. In the analyzed group of patients, stroke recurrence was the most significant risk factor for mortality after first-ever AIS, with a hazard ratio (HR) equal to 16.68 [30]. Since the etiology of AIS is multifactorial, many risk factors may also be related to the appearance of particular outcomes in both children and adults. Nevertheless, patients who suffer from AIS and have no risk factors for the disease are equally frequent. The data regarding the risk factors for AIS recurrence are not common and a number of them are retrospective. However, understanding the predictors (clinical, metabolic, or genetic) for the occurrence of subsequent ischemic stroke may be helpful in building strategies for secondary stroke prevention.

The aim of the present literature review was to discuss and compare the available data regarding the frequency of AIS recurrence in pediatric patients and in groups of young adults, as well as to assess the possible risk factors for recurrent stroke in children and young adults. We also followed data on the secondary prevention of AIS recurrence, both in children and young adults.

## 2. Review Methodology

We searched PubMed, Scopus, and Embase using combinations of the following keywords: “arterial ischemic stroke”, “ischemic stroke”, “stroke”, “recurrent”, “stroke recurrence”, “children”, “pediatric”, and “young adults” (last search early November 2019). As various definitions of “young adults” are available in the literature, we eventually took into account the articles analyzing adult patients younger than 55 years old and pediatric populations aged >one month to ≤18 years. In the present literature review, we included the results analyzing patients suffering from arterial ischemic stroke. For some data on AIS, its recurrence was discussed together with TIA, so we also included the papers. We excluded data on hemorrhagic stroke and cerebral sinuvenous thrombosis (CSVT), as well as perinatal and neonatal stroke. Finally, we discussed the results which we believed were the most interesting or relevant.

## 3. Prevalence of AIS Recurrence

### 3.1. In a Pediatric Population (Aged >1 Month to ≤18 Years)

Stroke and transient ischemic attack (TIA) recurrence occurs in a pediatric population with different frequencies, mainly dependent on the kind and number of AIS risk factors found in the patient [25,31]. AIS recurrence is another reason for severe neurological deficits increasing the ones caused by the first brain ischemia and mortality [25,32]. One of the major obstacles to the proper estimation of the frequency of recurrent AIS is the length of the follow-up period, which can vary significantly among studies.

The lowest frequency (2.7%) of recurrent AIS in children was reported by Steinlin et al. [33], who performed a study of patients from different locations situated far from each other (Switzerland and Australia). In addition, the number of patients was rather low. Per et al. reported a 7% frequency of AIS recurrence, with a median time of 6 months (the distribution interval between the recurrent episodes of AIS was 1–36 months) [34]. This level of prevalence was confirmed by Sultan et al. in a multicenter study including pediatric patients with stroke from 72 sites in 20 countries [35]. In this study, recurrent events comprised symptomatic AIS, silent AIS, or TIA [35]. A study based on pediatric patients from the USA demonstrated a 10.3% stroke recurrence, including 4% for anterior circulation AIS and 19% for posterior circulation AIS [36]. Almost 91% of these patients experienced recurrence within the first 6 months after AIS, whereas 45.5% of cases recurred within the first month [36]. A higher prevalence of recurrent stroke was noted by deVeber et al. [29] and Sfaihi et al. [37], namely 17.9% and 18% from 1 day to 136 months after the first stroke, respectively. In a group of 84 AIS children [38], the median interval to recurrence was 2.3 months and in most observed patients (77%), recurrence occurred within the first 6 months after stroke. Within 5 years of follow-up, AIS recurred in 13 pediatric patients (15.5%), whereas recurrence including TIA was present in 29% of patients (24 out of 84 children) [38].

In a study by Fullerton et al. [39], 42 out of 354 patients (11.86%) exhibited a recurrence of AIS. The cumulative rate of the first recurrent stroke was 6.8% at one month and 12% at one year [39]. The highest frequency of recurrent AIS was observed in Jordanian children after their first ever stroke (46%) in the follow-up, ranging from 1 month to 9 years (median follow-up of 2 years). However, the whole group of analyzed patients was extremely small, which may have influenced the results in a positive or negative way [40]. Surprisingly, no stroke recurrence was demonstrated in a population of 78 children with AIS from Katowice, Poland, during the follow-up, ranging from 1 month to 10 years [41]. The analyzed group of Polish pediatric patients consisted, among others, of children with heart disease treated with antithrombotics for prophylaxis. Moreover, no patients with diagnosed sickle cell disease (SCD) and Moyamoya disease were enrolled in the study. These facts may be an explanation for the lack of AIS recurrence in this group.

On the other hand, in a study performed using an international multicenter stroke database on children with Moyamoya disease, stroke recurrence was 20% over a median follow-up of 13 months, and 9% of patients had multiple recurrences [42].

Summarizing the data on the prevalence of recurrent stroke in children, the studies concerning this problem present groups of children which differ to a great extent in terms of the number of analyzed patients, their age at stroke onset, their ethnicity, the follow-up period, the etiology, and the stroke lesion location (anterior vs. posterior brain circulation), as well as secondary prophylaxis. For these reasons, an analysis of the data is very difficult and clear information on the number of stroke recurrences in pediatric populations remains largely undefined. Table 1 summarizes the data on the frequency of stroke recurrence in different pediatric populations.

### 3.2. In Young Adults (Aged 15–55 Years Old)

For a USA population, Kleindorfer et al. [43] reported a significant decrease in stroke incidence, but only among whites. Simultaneously, another study of this research team indicated that the proportion of all strokes under the age of 55 years increased from 12.9% to 18.6% within a decade [16]. Moreover, a study based on over 4000 young Danish patients (aged 15–30 years) showed rising hospitalization rates for first-ever stroke or TIA [44].

The prevalence of recurrent stroke in young adults may differ between populations. In a group of 428 patients with first-ever stroke, recruited from 46 hospitals in Baltimore City, five central Maryland counties, and Washington, DC, recurrent AIS was demonstrated in four cases (0.93%) during a study period from 1988 to 1991 [45]. A low frequency of stroke recurrence (2%) was found in a study from Rome, Italy, although it was carried out on a small group of patients with AIS (*n* = 150) within a mean follow-up period of 41.9 months [12]. In a study by Marini et al. [46], performed for patients recruited in seven neurology centers in Italy from April 1984 to March 1988, recurrent stroke occurred in 10 patients (3.29%), with a mean annual incidence rate of 2.36% higher in patients with mixed atherothrombotic and cardioembolic etiology than in patients included in the other diagnostic groups. A comparable group in terms of the number of patients to those studied by Renna et al. [12] was analyzed by Goeggel Simonetti et al. [47]. However, the authors demonstrated a higher frequency of recurrent cerebrovascular events (i.e., in seven patients (5%)), with a median follow-up period of 6.9 years. In a study by Li et al. [22], based on almost 1400 young adults from Northern China, 6.7% patients experienced AIS recurrence; the average duration after the first onset of stroke was 338.7 days. A similar frequency of stroke recurrence (6.26%) was demonstrated in a study by Pezzini et al. [48], who reported recurrent AIS in 32 patients. A study performed in patients with AIS from the Department of Neurology of the University of Iowa revealed a 9% frequency of recurrent stroke, with a mean interval between the initial and recurrent stroke of 4.7 years [49]. Among Estonian stroke patients, recurrence was observed in 11.5% of patients [50]. In comparison, a study by Aarnio et al. [30] demonstrated 13.6% cases suffering from recurrent stroke in a large group of almost 1000 Finnish patients. In one of the largest study groups, consisting of young Swedish patients, in whom the AIS onset occurred between 1987 to 2006 (over 17,000 patients), the prevalence of recurrent stroke within this period was 14.2% [51]. In turn, a study by Schellekens et al. demonstrated that the frequency of recurrent events in patients from The Netherlands was almost 19% (recurrent AIS was the most frequent) [52].

On the contrary, in Chinese patients with first-ever stroke, an almost two-fold higher frequency of stroke recurrence in comparison to a Finnish study was demonstrated within a mean follow-up period of over five years [53]. Similarly, a high prevalence of recurrent stroke (25%) was found in a Spanish population, with a mean period of 6.5 years between the initial stroke and the first recurrence [54]. The annual stroke recurrence rate during the first year was 3.6%, with a tendency to fall to 1.7% in subsequent years. The authors also observed that 60% of those patients had just one episode of recurrence, whereas others had more than one. In addition, 16% of patients died as a result of the recurrence [54].

Table 2 summarizes the data on the frequency of stroke recurrence in various populations of young adults.

## 4. Risk Factors for Recurrent AIS in a Pediatric Population

Arteriopathy, vasculopathies, and some genetic polymorphisms are among risk factors which may increase the risk of recurrent stroke in children. Since the etiology of AIS is multifactorial, the data also indicate that several risk factors may influence the risk of its recurrence. The study by Lanthier et al. [55] demonstrated that children with AIS and multiple risk factors were at a greater risk of recurrent AIS than children with only one risk factor.

### 4.1. Arteriopathies

AIS recurrence in a pediatric population may be related to the presence of arteriopathies. Focal cerebral arteriopathy of childhood (FCA) has been defined as stenosis revealed by brain vasculature imaging, not related to other specific etiologies, i.e., Moyamoya disease and syndrome, dissection, post-varicella arteriopathy, sickle cell disease, vasculitis, or post-radiation vasculopathy [27]. Previously, transient cerebral arteriopathy (TCA) was defined as transient brain middle and/or large artery stenosis; since 2009, it has been included in the definition of FCA [27,56]. The clinical presentation of FCA is acute and monophasic; the most important risk factor for FCA is a recent upper respiratory tract infection (URI) without any specificity to time between the infection onset and stroke onset, especially in early school-age children [6,27].

FCA causes a 5-fold increase in the risk of recurrent stroke in comparison with idiopathic AIS [39]. The study by Fullerton et al. [39] did not confirm that other risk factors, i.e., a low socioeconomic status, recent infection, and under-vaccination, are predictors of recurrent AIS in children. At an early stage, FCA may have a tendency to progress, leading to brain ischemia and its recurrence. Another study by Fullerton et al. demonstrated the link between presumed inflammation, FCA progression, AIS, and acute brain ischemic recurrence [57]. The authors observed higher concentrations of high-sensitivity C-reactive protein, as well as serum amyloid A, in children with arteriopathic, but not cardioembolic, stroke, thus revealing that the presence of the first one increased the risk of recurrent AIS [57].

In 2012, CASCADE classification was created to identify, in each AIS pediatric patient at the disease onset, any anatomical abnormality within the brain and neck vessels and the heart [58]. The categories of anatomical abnormalities from 1 to 4 within the acute primary classification correspond to arteriopathies, as follows: 1—small vessel arteriopathy of childhood, 2—unilateral focal cerebral arteriopathy of childhood (FCA), 3—bilateral cerebral arteriopathy of childhood with collaterals (3a, fibromuscular dysplasia, Moyamoya; 3b, without collaterals), and 4—aortic/cervical arteriopathy (e.g., dissection) [58]. Other CASCADE categories are 5—cardioembolic, and then 6 and 7—other and multifactorial, respectively. The CASCADE categories from 1 to 4 may be, at follow-up, classified as progressive, stable, reversible, or indeterminate [58].

In 2018, British authors from the Great Ormond Street Hospital, London, identified 84 AIS children (mostly girls, with a mean age at onset of 4.1 years); the cases were classified according to the CASCADE criteria and followed for a maximum of four years (mean follow-up period was 2.4 years) [38]. As for the CASCADE types, 3a and 3b were significantly associated with the risk of AIS recurrence within the analyzed group of children [38]. In a German study, 57 out of 86 AIS children enrolled between 2004 and 2017 met the criteria of arteriopathic stroke (1–4 CASCADE classification) [25]. The median age at stroke onset was 7.9 years and the median time of the follow-up period was 2.1 years (maximum 4.4 years), which was similar to the British research scheme. Categories 2 and 3 of the CASCADE classification were statistically significant risk factors for stroke recurrence in this group of patients, whereas the patients with unilateral FCA showed early progress and the recurrence of brain ischemia (11 days) and the patients classified as the 3 CASCADE group presented late progress of arteriopathy (124 days) [25]. The above results have proven that CASCADE classification not only helps to unify the definitions and AIS patients’ descriptions, but may also be a useful tool for predicting the long-term outcome in pediatric stroke patients. Cases with types 2 and 3 are at a higher risk of vessel wall pathology progress, and, as a result, early or late stroke recurrence [25,38]. This may be useful and practical information for clinicians for more careful patient observation. Secondary prevention for patients with the CASCADE type 2 category is corticosteroid and antithrombotic treatment, even if the risk for recurrent stroke in these children is low [33,38]. In the group described by Stacey et al. [38], two out of 84 children (3a and 3b in the CASCADE classification) did not get any post-stroke treatment and both experienced recurrent AIS.

Until now, one of the confirmed risk factors for recurrent stroke in children has been the presence of vasculopathy, even when Moyamoya is excluded. In a study by deVeber et al. [29], AIS recurrence was significantly more common among children with vasculopathy than in children without vasculopathy (HR = 2.5).

Sickle cell disease (SCD) is one of the most common genetic reasons for anemia, mostly present in sub-Saharan Africa, the Middle East, and India and caused by a mutation in the hemoglobin gene (β subunit) in chromosome 11 [59]. SCD affects different organs and some of the most devastating problems in the course of the disease are central nervous system complications. Progressive arteriopathy mainly concerns the large arteries of the anterior brain vasculature (MCA, middle cerebral artery; ACA, anterior cerebral artery; ICA, internal carotid artery), with progressive narrowing of the vessels and new collaterals similar to Moyamoya vasculopathy. If Moyamoya (“puff of smoke” on digital subtraction angiography) appears in the course of SCD, it is defined as Moyamoya syndrome; in the CASCADE classification, it will meet the criteria of class 3a [58,59]. The clinical presentation of neurological complications during SCD is represented by both cognitive decline and silent cerebral ischemia (SCI), with radiological findings of brain infarction without clinical symptoms and clinically overt AIS. The last one is present in more than one third of SCD patients before the age of 18 years [60,61]. Stroke in SCD patients is a leading cause of disability and morbidity, the prevalence of AIS in children and young adults with SCD is 3.75%, and the peak of prevalence occurs at the age of 2 to 5 years [62]. Both Moyamoya syndrome and progressive vasculopathy in children with SCD are risk factors for recurrent silent and clinically overt ischemic strokes; the risk was estimated to be 12.7/100 patient-years. The indirect revascularization procedure is a proven method for reducing recurrent ischemic brain incidents in these patients [63].

### 4.2. Cardiac Defects

Pediatric patients with heart disease are at a high risk of stroke and its recurrence; congenital and acquired cardiological problems are “classical” risk factors for stroke in pediatric populations. In a study by Vazquez Lopez et al. [64], within a 15-year follow-up observation of all children hospitalized for heart disease, mostly congenital cardiomyopathy, 74 suffered a stroke; the age of the children at stroke onset varied from 1.6 months to 51.7 months (mean age of 11.7 months) and the mean age at evaluation was 8.9 years ± 4.4 years. Most children—up to 70% of the examined group—presented an unfavorable neurological outcome (sensimotor disability, as well as cognitive impairment); 20% of the children died; and in 10%, recurrent stroke was observed. On the other hand, in the study by de Veber et al. [31], the only statistically significant risk factor for AIS recurrence was arteriopathy, even if a new ischemic episode was observed in 11 children out of 15 with cardiac disease.

In a study by Rodan et al. [65], which was performed in children with congenital heart disease and AIS, a mechanical valve and a prothrombotic condition were independent risk factors for stroke recurrence. In turn, Per et al. [34] observed recurrent stroke in 7% of cases of the analyzed group, and infection and cardiac catheterization for mostly congenital heart diseases were the most important risk factors for AIS in this group.

### 4.3. Thrombophilia

According to an international study by deVeber at al. [29], the following prothrombotic risk factors, i.e., antithrombin deficiency, elevated lipoprotein (a), and the co-existence of more than one prothrombotic factor, were associated with the increase in AIS recurrence risk when adjusting for vasculopathy. The authors observed a single prothrombotic disorder in 269 children with AIS, whereas in 88 patients, more than one prothrombotic risk factor was detected. In addition, heterozygous antithrombin deficiency, high lipoprotein (a), high fibrinogen, and high fasting homocysteine were related to AIS recurrence [29].

### 4.4. Seizures in the Clinical Presentation of AIS

In a 16-year, prospective, national population-based study, i.e., the Canadian Pediatric Ischemic Stroke Registry by deVeber et al. [31] based on children with AIS recruited during the period from 1992 to 2001, several risk factors which could predict AIS recurrence were analyzed. The authors found that seizures in the clinical presentation of AIS were not risk factors for a poor outcome; the frequency of seizures was comparable in groups of children with a poor and good outcome (49% vs. 47%, respectively). In turn, a higher frequency of seizures was observed among children without recurrent AIS compared to those with recurrent AIS (40% vs. 21%, respectively). Therefore, clinical presentation without seizures was a predictor for stroke recurrence, with HR = 1.96 (*p* = 0.025) [31].

### 4.5. Genetic Polymorphisms

Recently, several reports indicated relationships between specific genetic polymorphisms and the recurrence of stroke in children. The genetic risk factors, due to the age of the patients, especially pediatric ones, may be of specific importance in the appearance of subsequent AIS. However, studies on the topic are not common and show analyses of various polymorphic variants. Most often, a reliable statistical analysis regarding the relation between a polymorphic variant and stroke recurrence is not possible in pediatric patients due to the fact that recurrent AIS only occurs in a few cases. Therefore, published data on the topic are difficult to interpret and require confirmation based on larger patient populations with AIS.

Coen Herak et al. analyzed 73 children with perinatal and childhood stroke and found no polymorphic variants of thrombotic factors, i.e., factor V (*FV*) Leiden, *FV* HR2, or factor II (*FII*) 20210G>A, among patients with recurrent AIS [66]. On the other hand, the authors found that the frequency of the *HPA* 2a/b genotype was over 2-fold higher in cases with recurrent childhood AIS than in cases with nonrecurrent childhood AIS. Additionally, 75% of patients with recurrent stroke had a combined *APOE* ε2ε3 and *ACE* I/D genotype, whereas in the pediatric group without recurrent stroke, it was only 6%. In this study, it was also demonstrated that one of the children with recurrent stroke was homozygous for methylenetetrahydrofolate reductase (*MTHFR*) 677C>T polymorphism [66]. In turn, in children with AIS from Lebanon, 677C>T polymorphism within the *MTHFR* gene was found to be present in the patients who suffered from recurrent stroke, as well as those who had multiple risk factors for AIS [67]. Previously, the T allele carrier-state of 677C>T polymorphism in the *MTHFR* gene was proved to increase the risk of AIS in children [68], in contrast to another common 1298A>C polymorphism in the *MTHFR* gene [69]. The polymorphic 677TT variant of the *MTHFR* gene was previously linked to a higher level of homocysteine, one of the biochemical risk factors for cerebro- and cardiovascular diseases, both in children and adults.

On the other hand, in a large cohort of almost 900 pediatric patients with first-ever AIS from Canada, the UK, and Germany, a subsequent AIS event was diagnosed in almost 18% of patients and the authors found that the presence of isolated mutations in *FV* at rs6025, as well as in *FII* at rs1799963, were not individually significantly associated with recurrent AIS [29]. In a study by Per et al. [34], *FV* Leiden mutation and homocystinuria were present in patients who experienced subsequent strokes.

## 5. Risk Factors for Recurrent AIS in Young Adults

Risk factors for recurrent stroke observed in children and older patients may differ. However, similar to the observation by Launthier et al. [55], a study by Pezzini et al. [48] based on young adults from Italy demonstrated that the risk of recurrence increased with an increasing number of traditional factors in comparison to patients with no risk factor (HR was 2.29 for subjects with one factor and 5.28 for subjects with two factors). In addition, a significant role of predisposing genotypes was also demonstrated [48]. However, the data on risk factors for stroke recurrence in young adults are not as common as in older patients.

### 5.1. Hypertension

Hypertension was demonstrated to be a risk factor for stroke in young adults [70]. In a study by Putaala et al. [71], hypertension was present in 39% of patients with AIS. It was demonstrated that recurrent ischemic stroke occurred in 13.6% of young patients with well-documented risk factors, and in only 4.7% of young patients without well-documented risk factors. The presence of ≥4 well-documented risk factors was independently associated with a higher risk for recurrent ischemic stroke [71].

The analysis of traditional risk factors by Pezzini et al. [48] revealed that hypertension and a family history of AIS under the age of 45 years were significantly related to the recurrence of vascular events in young adults. The authors demonstrated a significant difference in the prevalence of patients with hypertension in the group having recurrent vascular events compared to the whole analyzed group of patients (31.5% vs. 20.7%; HR 2.36).

Hypertension was also found to be a risk factor for recurrent stroke in Estonian patients. It was present in almost 69% of AIS cases and in 53% of cases with first-ever stroke [50]. In a study by Xu et al. [72], hypertension was also associated with an increased risk of recurrence in a large cohort of Chinese patients with stroke. However, the age range of the patients was very wide, from 19 to 97 years. In addition, the authors made an important observation that controlling hypertension significantly reduced the recurrent risk [72].

### 5.2. Diabetes Mellitus

Diabetes mellitus is one of the established risk factors for stroke recurrence in young adults. Previous data indicated that patients aged 18–50 years with TIA or ischemic stroke and with diabetes and impaired fasting glucose (IFG) were more likely to experience a vascular event compared to subjects with normal fasting glucose [73]. However, in the case of risk for the recurrence of stroke, it was similar for patients with incident diabetes and IFG compared to those with normal fasting blood glucose values. At follow-up, almost 13% of patients with IFG had at least one stroke compared to 8% of patients with normal fasting glucose [73].

Among cases of patients from Estonia suffering from AIS recurrence, diabetes mellitus and peripheral artery disease were observed with a significantly high frequency compared to the group of patients with first stroke (18.8% vs. 8.7% and 5.5% vs. 1.1%, respectively). Additionally, Schneider et al. [50] demonstrated that patients with recurrent stroke were older than those with first-ever stroke and recurrent AIS was more frequently caused by large-artery atherosclerosis.

An earlier study by Naess et al. [74] including 232 patients with first-ever stroke demonstrated that diabetes mellitus is a factor associated with subsequent vascular events. Other risk factors observed in the study were as follows: myocardial infarction, angina pectoris, intermittent claudication, and smoking, However, in this study, myocardial infarction was analyzed jointly with recurrent stroke as a vascular event. The authors also observed that patients with no risk factor had a low frequency of recurrent vascular events.

An analysis based on 121 Chinese patients with Moyamoya disease and first-ever stroke (aged 18–45 years), who underwent revascularization after the acute phase of initial stroke, proved that diabetes is an independent predictor for stroke recurrences (HR 6.76) [75]. Diabetes was also significantly associated with unfavorable functional outcomes in this study (odds ratio (OR) of 7.87).

### 5.3. Dyslipidemia

Data on relations between lipid abnormalities and the risk of stroke recurrence in young adults are most often contradictory. In older adults, lipid abnormalities were previously associated with a higher risk of ischemic stroke recurrence in patients with a large-artery atherosclerosis subtype of stroke [76]. In young adults with stroke from the USA, hypercholesterolemia was associated with a higher cumulative risk for both cardiac events and recurrent stroke [77]. Li et al. demonstrated that abnormal lipid metabolism was associated with AIS recurrence, but only in univariate, and not multivariate, analysis [22]. In turn, Pezzini et al. [48] did not observe a significant difference in the prevalence of patients with hypercholesterolemia in the group with recurrent vascular events compared to the whole analyzed group of patients (27.4% vs. 26%) [49]. This finding was confirmed based on a three-times larger group of patients (*n* = 1867), also by Pezzini et al. (24.4% of patients with hypercholesterolemia in a group without recurrence vs. 28.2% in a group with recurrence) [78].

Atherogenic dyslipidemia increased the stroke recurrence risk among stroke patients of the large-artery atherosclerosis subtype (HR 2.79), but not in all stroke patients (HR 1.69) [76]. The mean age of the patients with dyslipidemia and AIS was 58.77 years. It was also found that the stroke recurrence rate was significantly higher in patients with atherogenic dyslipidemia than in those without this condition (20.3% vs. 11.9%).

In a study by Schneider et al. [50] in Estonia, both young patients with first-ever stroke and patients with recurrent stroke showed a high prevalence of dyslipidemia (45.5% vs. 47.9%, respectively). However, it was not a risk factor for recurrence.

### 5.4. Thrombophilia

Studies analyzing thrombophilic factors with regard to stroke recurrence are scarce. In the study by Schellekens et al. [52], no relations between the presence of a prothrombotic factor or some recent infections and an increased risk for any recurrent ischemic event or recurrent cerebral ischemia in young patients with cryptogenic stroke were demonstrated after a mean follow-up period of 8.9 years. Similarly, no differences in haemostatic factors (i.e., fibrinogen and D-dimer) were observed between patients with and without recurrent acute cerebral ischemia in patients from Slovenia aged under 45 years [79]. The case of a young woman with recurrent TIA demonstrated an elevated level of fibrinogen, as well as homocysteine, after methionine loading [80].

### 5.5. Smoking

Cigarette smoking is one of the modifiable risk factors for both primary and secondary stroke prevention in young adults.

In young Estonian patients with AIS, the prevalence of smokers was similar between first-ever stroke and recurrent stroke subgroups (34.7 vs. 28.1, respectively) [50]. Therefore, smoking cessation may have a low efficiency in secondary prevention against recurrent stroke in the young. In turn, smoking was a risk factor for a subsequent vascular event [74]. In this study, the prevalence of smokers in the group with a subsequent vascular event was significantly higher than in the group without these events (78.6% vs. 58.2%, respectively) [74]. Similarly, in a study by Pezzini et al. [78], current smokers were significantly more common among patients with recurrent vascular events than in the group without recurrence (37% vs. 46.6%, respectively). In turn, in young Chinese patients, smoking was only related to stroke recurrence in univariate analysis, while multivariate analysis did not confirm this finding [22].

### 5.6. Substance Abuse

One of the most commonly abused drugs in the USA is cocaine. In a study by Cheng et al. [81], a significant association between acute cocaine use and the risk of AIS in young adults was demonstrated after adjusting for smoking, alcohol consumption, and hypertension (OR = 5.7). As cocaine may increase the risk of AIS, the discontinuation of its use may prevent recurrent stroke [82]. A case of a 39-year-old man who developed occlusion of the frontopolar branches of the left middle cerebral artery 1 h after intravenous cocaine use was described by Sauer in 1991 [83]. Eleven days later, the man developed occlusion of the superior division of the right middle cerebral artery.

### 5.7. Genetic Polymorphisms

As for genetic analysis in relation to stroke recurrence in young adults only, the data are poor. The study by Ou et al. [84] demonstrated that the wild-type G allele of -174G>C polymorphism within the interleukin-6 gene significantly increased the risk of stroke recurrence in comparison to the C allele in young adult patients with moderate internal carotid artery stenosis. In turn, the study by Pezzini et al. [48], which was performed in a large cohort of over 500 patients with AIS younger than 45 years, three of the commonly analyzed polymorphisms (the 20210A variant of the prothrombin gene, the 1691A variant of the *FV* gene, and the TT677 genotype of the *MTHFR* gene) were assessed in relation to the recurrence of ischemic events. The authors observed that the risk of recurrence increased with an increasing number of predisposing genotypes (from HR equal to 1.96 for cases with one genotype to HR equal to 3.83 for cases with two genotypes).

Almost 40% of ischemic strokes in adults are cryptogenic and the prevalence of persistent foramen ovale (PFO) is two-fold higher in these patients than in healthy patients. In turn, the most frequent cause of embolism in cryptogenic stroke associated with PFO is deep venous thrombosis. One of the earliest studies analyzing genetic variants in the recurrence of thrombotic events was performed by de Stefano et al. [85]. The authors demonstrated that in simultaneous carriers of *FV* Leiden and the 20210G>A polymorphism in the prothrombin gene, the risk of recurrent deep venous thrombosis after the first episode was significantly increased.

Despite the fact that the results of genetic analyzes regarding the onset of stroke recurrence are inconclusive, these are undoubtedly important data which should be considered in the interaction with other, non-genetic risk factors in estimating the risk of recurrence.

### 5.8. Other Predictors

Various other predictors for recurrent ischemic stroke have been reported. Previously, Pezzini et al. [78] demonstrated migraine with aura as a risk factor for recurrence. The Chinese study, which included a sizeable group of young patients, revealed, in a univariate analysis, that AIS recurrence was associated with atrial fibrillation, TOAST type in patients with an unclear cause, and the National Institutes of Health Stroke Scale (NIHSS) score at admission. However, the multivariate analysis only confirmed the NIHSS score to be a predictor of recurrent stroke [22]. In a study by Nedeltchev et al. [28], recurrent stroke was significantly associated with a previous history of TIA. Additionally, the authors observed that age, sex, stroke risk factors other than previous TIA, and stroke etiology, as well as stroke subtypes, were not related to the risk for recurrence.

In turn, among Finnish young adults with stroke, patients with an index stroke caused by high-risk sources of cardioembolism had the highest risk of any subsequent cardiovascular events (HR 3.7), whereas patients with large-artery atherosclerosis had a higher risk of recurrent stroke (HR 2.7) than patients with stroke of an undetermined etiology [30].

In addition, independent predictors for vascular event recurrence in young adults were a familial history of stroke, aPL, and discontinuation of antiplatelet and antihypertensive medications in an Italian population [78].

**Figure 1:**
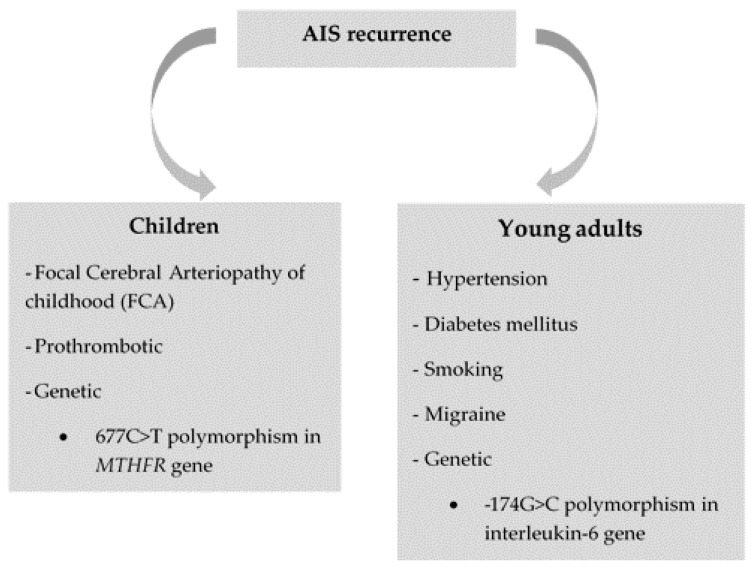
Potential risk factors for stroke recurrence identified in different pediatric and young adult populations.

Figure 1 lists the potential risk factors for stroke recurrence in pediatric patients and young adults.

## 6. Secondary Prevention of AIS

### 6.1. In Pediatric Patients

The proposition of secondary prophylaxis in AIS children was published in 2007 [33]. In the group of 73 children diagnosed with AIS and FCA, treatment with a combination of corticosteroids and antithrombotics (CAT) was administered, whereas 52 children received only antithrombotic treatment (AT). Stroke recurrence was observed in each group, but the complete stenosis resolution was found in 81% of CAT patients vs. 59% of AT patients [33]. Even though, at the end of the follow-up, the number of recurrences in these children was the same in both subgroups, the complete resolution of vascular stenosis was more commonly seen in the CAT group, which exhibited a good correlation with a longer AIS recurrence prognosis. For this reason, the proposition of CAT after the first episode of brain ischemia in children seems to be an attractive therapeutic tool, even though a study of a larger group of patients is still needed. As FCA is presumed to be of inflammatory etiology, the proposition of immunosuppressants as secondary prophylaxis was published [86].

In a study by Stacey et al. [38], two patients with the highest risk of AIS recurrence (3a and 3b, according to the CASCADE classification) did not receive any secondary prophylaxis and both of them showed the recurrence of brain ischemia. In total, 82 out of 84 patients from the presented group received antiplatelet and/or antithrombotic therapy (only antiplatelet in 68 children, and both in seven children); the number of recurrences was 155 within 60 months [38]. Even if antithrombotic therapy is used for secondary AIS prevention in children with specific arteriopathy subtypes, consisting of 3A and 3B of the CASCADE classification, the risk of recurrence is very high [38]. Considering the results of Steinlin et al. [33] and Stacey et al. [38], the most important cause of pediatric AIS recurrence is the type of arteriopathy in the CASCADE classification, where some of the subtypes (FCA) are not so strongly associated with recurrent brain ischemia, whilst other subtypes, 3A and 3B, lead to recurrence, even if secondary prophylaxis is administered.

Preventive treatment for children with SCD is specific for the disease itself, but also for a particularly high risk of stroke in SCD (TAMMV, time-averaged mean of maximum velocities, of ICA or MCA ≥ 200 cm/s). In these patients, chronic red cell transfusions and/or hydroxyurea may be administered as alternative therapy; for the first one, serious side-effects (iron overload, transfusion reactions, infection, alloimmunization) may be the cause of switching to hydroxyurea treatment [59]. Despite receiving secondary prophylaxis with transfusions, approximately 20% of SCD patients had stroke recurrence [87]. Bone marrow transplantation (BMT) in SCD patients below the age of 16 observed in the mean time of 7 years revealed that these patients were protected against stroke and even experienced vasculopathy stabilization [88]. Antithrombotic and especially antiplatelet therapy in SCD children is rather an adjuvant therapy. More promising ways of treating cerebral vasculopathy in SCD patients are surgical procedures like encephaloduroarteriosynangiosis (EDAS) and plial synangiosis [89].

MELAS (mitochondrial encephalopathy, lactic acidosis, and stroke-like episodes) pediatric patients are at a high risk of recurrent stroke-like episodes (in radiological presentation, the locations of stroke areas do not correspond to a typical brain vascular distribution); in 80% of patients, the disease is caused by mutation 3243A>G within the *MT-TL1* gene. The first symptoms occur at the age of 2 years and include muscle weakness, a short posture, learning difficulties, migraine and consciousness disturbances, seizures, hearing impairment, and neuropathy of the peripheral nerves, as well as diabetes and cardiomyopathy. In secondary prophylaxis, after the first ischemic episode, to prevent recurrent stroke-like episodes, arginine should be administered at a 150–300 mg/kg/daily oral dose [90].

In a group of 135 Canadian children with AIS and congenital heart disease analyzed by Rodan et al. [65], 19 patients had stroke recurrence (median follow-up period was 2.15 years). Of them, seven (37%) were not administered with any antiplatelet or antithrombotic medications, three took an anticoagulant at a subtherapeutic level, and the remaining children (*n* = 9) with stroke recurrence took an antiplatelet agent or an anticoagulant agent at therapeutic levels, or both. At the last follow-up, 32% of the patients without stroke recurrence were on acetylsalicylic acid (ASA), 15% were on warfarin, 11% were on low molecular weight heparin, and 39% did not take any antiplatelet or antithrombotic medication. In this group, almost 53% of children with recurrent AIS did not receive effective antithrombotic treatment at the time of recurrence [65].

### 6.2. In Young Adults

Since secondary stroke prevention aims to reduce the risk of subsequent AIS, it is important to know the etiologic mechanism of the initial stroke, as well as accompanying risk factors. Together with antiplatelet or anticoagulant therapy, secondary prevention should include the treatment of vascular risk factors (arterial hypertension, hyperlipidemia, diabetes mellitus, and cardiac disease) and/or surgical procedures. However, knowledge on medications used in secondary prevention in young adults is limited. It has therefore been noted that the long-term use of recommended medications raises the greatest problem in preventing secondary stroke, since approximately 30% of stroke patients discontinued one or more drugs within 1 year of hospital discharge [91].

According to the European Stroke Initiative Recommendations for Stroke Management guideline, aspirin should be an antiplatelet drug of first-choice in secondary prevention [92]. In patients with ASA intolerance, clopidogrel at a dose of 75 mg/day may be considered as the first-choice treatment. Where possible, combined aspirin and dipyridamole (25/200 mg twice daily), or triflusal alone, may be used. The combination of ASA and clopidogrel is only recommended in secondary stroke prevention in the case of the coincidence of stroke and recent myocardial infarction or status post-coronary stenting. In cardioembolic AIS, prevention is based on the use of oral anticoagulants [93]. In patients with non-embolic ischemic stroke or TIA, antiplatelet oral agents are more preferable to reduce the risk of recurrent stroke and other cardiovascular events than anticoagulant therapy [94].

In patients with stroke recurrence, novel oral anticoagulants (NOAC; thrombin and factor X inhibitors) can be used for the secondary prevention of cardioembolic stroke, despite appropriate treatment with warfarin [95]. In atherothrombotic IS, antiplatelet therapy and revascularization procedures reduce the risk of recurrence in selected cases of ipsilateral carotid stenosis [93].

A specific role of statins in young adults with AIS has also been discussed. Their benefit in patients with predominantly nonatherosclerotic stroke etiologies is not clear. Putaala et al. [96] demonstrated that patients taking statins at any time during follow-up had a lower risk of outcome events (i.e., stroke, myocardial infarction, other arterial thrombosis, revascularization, or vascular death; HR 0.23). The authors observed that 20% of events occurred among patients who had never been on statins, none occurred among patients with continuous statins, and 11% occurred among patients with discontinuous statins [96].

## 7. Conclusions

Understanding the risk factors for the recurrence of arterial ischemic stroke in the young may help to identify the mechanisms of AIS, especially in children, and in some cases, may contribute to disease prevention. Current knowledge on risk factors for recurrent AIS suggests that multiple risk factors may be related to stroke recurrence, both in children and young adults. Risk factors for stroke recurrence differ between child and young adult populations, which is clearly demonstrated in the present literature review. The best documented risk factors for recurrent AIS in children are arteriopathies, especially FCA, which significantly increase the risk of recurrence. A proposal for secondary prevention in children with FCA to avoid subsequent strokes has even been demonstrated. Treatment of a combination of aspirin and corticosteroids allows for the total resolution of vessel wall stenosis in FCA, and, in this way, this therapy may decrease the risk of AIS recurrence. On the other hand, even if antithrombotic medication is used, pediatric AIS recurrence remains a significant problem, especially in children with specific arteriopathy subtypes of the CASCADE classification, i.e., 3A and 3B.

Risk factors for recurrent stroke such as diabetes mellitus, hypertension, or lipid abnormalities, have been highlighted in young adults. Undoubtedly, many risk factors, including genetic ones, have an impact on AIS etiology, as well as on the presence of its subsequent consequences, including recurrence. Understanding the links between the presence of a specific risk factor and the occurrence of a post-stroke outcome may be of particular importance for practical reasons. Establishing predictors of stroke recurrence may allow for more effective treatment, and may thus reduce the number of cases.

## Figures and Tables

**Table 1:** Prevalence of arterial ischemic stroke (AIS) recurrence in different pediatric populations.

Study	Type of the Study	Population	Age at Time of First AIS	No. of Analyzed Patients/No. of Patients with Recurrent AIS	Time of the Follow-Up	Frequency of Recurrent AIS
Bohmer et al. [25]	Retrospective	Patients admitted to Department of Pediatrics, University Hospital of Muenster, Germany	>29 days and <18 years of age	86/21	Median: 2.1 (interquartile range: 0.7–4.4 years)	21%
deVeber et al. [29]	Retrospective	Patients from Canada (Toronto), Germany (Kiel-Lübeck/Münster), and the UK (London/Southampton)	1 month to 18 years	894/160	median: 35 months (minimum-maximum: 1–256 months)	17.9%
Steinlin et al. [33]	Retrospective	Patient cohorts with FCA from Switzerland and Melbourne (Australia)	1 month to 18 years	73/2	(7 months to 31 months)	2.7%
Per et al. [34]	Retrospective	Turkish pediatric patients	1 month and 16 years	130/9	5 months to 11 years	7%
Sultan et al. [35]	Cross-sectional analysis	Patients from 72 sites in 20 countries	At least 28 days of age and less than 18 years	1652/95	from January, 2003 through April, 2012	5.75% (TIA, AIS, and silent AIS)
Uohara et al. [36]	Retrospective	Patients recruited at The Children’s Hospital of Philadelphia, USA	Between 29th day of age and 17.99 years	107/11	Median: 20.9 months (interquartile range: 8.7–40.4 months)	10.3%
Stacey et al. [38]	Retrospective	Patients admitted to Great Ormond Street Hospital, UK	>28 days and <18 years of age	84/24	Median: 2.4 years (interquartile range: 1.5–4.0 years)	AIS: 15%; both AIS and TIA: 29%
Fullerton et al. [39]	Retrospective	Children with AIS from 37 international centers	aged 29 days through 18 years	354/42	Median: 2.0 years (interquartile range: 1.0–3.0)	11.9%
Masri et al. [40]	Retrospective	Pediatric patients from Child Neurology Clinic at Jordan University Hospital (Jordan)	1 month to 13 years (median: 5 years).	24/11	Period ranged from 1 month to 9 years	46%

**Table 2:** Prevalence of AIS recurrence in different populations of young adults.

Study	Type of the Study	Population	Age at Time of First AIS	No. of Analyzed Patients/No. of Patients with Recurrent AIS	Time of the Follow-Up	Frequency of Recurrent AIS
Renna et al. [12]	Retrospective	Patients hospitalized at the stroke unit of Policlinico Gemelli of Rome, Italy	Younger than 50 years (mean age: 41 ± 8.0)	150/3	Mean: 41.9 months	2%
Li et al. [22]	Retrospective	Patients from Northern China	18–45 years	1395/94	At least one year	6.7%
Aarnio et al. [30]	Retrospective	Patients hospitalized at the Department of Neurology, Helsinki University Central Hospital, Finland	15–49 years	970/132	Mean: 10.2 ± 4.3 years	13.6%
Marini et al. [46]	Prospective	Patients recruited at seven departments of neurology (Florence, Genoa, L’Aquila, Milan, Padua, Parma, and Rome)	15–44 years	304/10	Average: 96 months (range: 62–124)	3.3%
Goeggel Simonetti et al. [47]	Prospective cohort study	Cohort was based on two registries: the Swiss Neuropediatric Stroke Registry and the Bernese stroke registry	16.1–45 years	154/7	Median: 6.9 years (interquartile range: 4.7–9.4)	5%
Pezzini et al. [48]	Retrospective	Patients form three Italian centers	Younger than 45 years	511/32	Mean: 43.4 months	6.3%
Kapelle et al. [49]	Retrospective	Patients hospitalized at the Division of Cerebrovascular Diseases of the Department of Neurology of the University of Iowa, USA	15–45 years	253/23	15 years, from 1st July 1977 to 1st January 1992	9%
Sneider et al. [50]	Retrospective	Patients treated in Tartu University Hospital and North Estonia Medical Centre	18–54 years	837/96	From January 2003 to December 2012	11.5%
Giang et al. [51]	Retrospective	Swedish patients	18–54 years	17149/2432	From 1987 to 2006	14.2%
Schellekens et al. [52]	Prospective	Patients admitted to the Radboud University Medical Centre Nijmegen, The Netherlands	18–50 years	415/29	Mean: 8.9 years	18.6%
Huang et al. [53]	Retrospective	Patients recruited from XuanWu Hospital, China	18–45 years	350/89	Average: 5.8 ± 3.2 years	25.4%
Varona et al. [54]	Retrospective	Patients admitted to the Neurology Department of the University Hospital, Madrid, Spain	15–45 years	240/61	Mean: 12.3 years	25%
